# Supplementary material for: Genetic Characterization of the Local Pirenaica Cattle for Parentage and Traceability Purposes
Source: Animals (Basel). 2020 Sep 5;10(9):1584. doi: 10.3390/ani10091584 (PMC7552125; doi:10.3390/ani10091584)
Supplement: Supplementary file 1 [file animals-10-01584-s001.pdf]

animals

Supplementary Materials: Genetic Characterization of the Local Pirenaica Cattle for Parentage and Traceability Purposes

David Gamarra [1], Masaaki Taniguchi [2], Noelia Aldai [3], Aisaku Arakawa [2], Andres Lopez-Oceja [1] and Marian M. de Pancorbo [1,*]

[1] Biomics Research Group, Lascaray Research Center, University of the Basque Country (UPV/EHU), 01006 Vitoria-Gasteiz, Spain; davidgamarrafdz@gmail.com (D.G.); andreslopezoceja@gmail.com (A.L.-O.)
[2] Animal Genome Unit, Institute of Livestock and Grassland Science, National Agriculture and Food Research Organization (NARO), Tsukuba 305-0901, Japan; masaakit@affrc.go.jp (M.T.); aisaku@affrc.go.jp (A.A.)
[3] Lactiker Research Group, Department of Pharmacy and Food Sciences, University of the Basque Country (UPV/EHU), 01006 Vitoria-Gasteiz, Spain; noelia.aldai@ehu.eus
* Correspondence: marian.mdepancorbo@ehu.eus

Table S1. Microsatellite markers ordered according to the highest F_{ST} value in Pirenaica breed.

Ranking	Marker	F_{ST}
1	ILST005	0.289
2	HEL05	0.195
3	ETH185	0.177
4	TGLA126	0.159
5	BM2113	0.146
6	HAUT27	0.136
7	INRA037	0.136
8	TGLA122	0.134
9	HAUT024	0.129
10	CSSM66	0.121
11	SPS115	0.120
12	HEL13	0.118
13	INRA063	0.116
14	ETH225	0.115
15	ETH152	0.103
16	HEL09	0.094
17	TGLA53	0.086
18	ETH010	0.073
19	ILSTS006	0.066
20	MM12	0.057
21¶	INRA023	0.051
22	ETH03	0.046
23	INRA032	0.046
24	TGLA227	0.045
25	INRA035	0.031
26	BM1818	0.023
27	CSRM60	0.018
28	HEL01	0.016
29	INRA005	0.012
30	BM1824	0.002

A minimum of 21 markers are necessary for trustworthy assignments (≥ 95 %) in Pirenaica breed.
